# Redesign of a Piston for a Diesel Combustion Engine to Use Biodiesel Blends

**DOI:** 10.3390/ma14112812

**Published:** 2021-05-25

**Authors:** Jorge Israel Noriega Lozano, Juan Carlos Paredes Rojas, Beatriz Romero Ángeles, Guillermo Urriolagoitia Sosa, Belén Alejandra Contreras Mendoza, Christopher Rene Torres San Miguel, Georgiy Polupan, Guillermo Manuel Urriolagoitia Calderón

**Affiliations:** 1 Instituto Politécnico Nacional, Escuela Superior de Ingeniería Mecánica y Eléctrica, Unidad Profesional Adolfo López Mateos, Gustavo A. Madero, Col. Lindavista, Ciudad de México C.P. 07738, Mexico; jnoriegalozano@gmail.com (J.I.N.L.); BROMEROA@ipn.mx (B.R.Á.); guiurri@hotmail.com (G.U.S.); bele02mendoza@gmail.com (B.A.C.M.); ctorress@ipn.mx (C.R.T.S.M.); gpolupan@ipn.mx (G.P.); gurriolagoitiac@ipn.mx (G.M.U.C.); 2 Centro Mexicano para la Producción más Limpia, Instituto Politécnico Nacional, Acueducto de Guadalupe S/N, La laguna Ticomán, Ciudad de México C.P. 07340, Mexico

**Keywords:** biodiesel, piston, diesel engine, numerical simulation, finite element method

## Abstract

Biofuels represent an energy option to mitigate polluting gases. However, technical problems must be solved, one of them is to improve the combustion process. In this study, the geometry of a piston head for a diesel engine was redesigned. The objective was to improve the combustion process and reduce polluting emissions using biodiesel blends as the fuel. The methodology used was the mechanical engineering design process. A commercial piston (base piston) was selected as a reference model to assess the piston head’s redesign. Changes were applied to the profile of the piston head based on previous research and a new model was obtained. Both models were evaluated and analyzed using the finite element method, where the most relevant physical conditions were temperature and pressure. Numerical simulations in the base piston and the new piston redesign proposal presented similar behaviors and results. However, with the proposed piston, it was possible to reduce the effort and the material. The proposed piston profile presents adequate results and behaviors. In future, we suggest continuing conducting simulations and experimental tests to assess its performance.

## 1. Introduction

The use of combustion machines began in the 18th century. The need to expel water from mines led to the steam engine’s development, which used wood, coal, or oil as fuel [1]. Inconveniences such as extreme working conditions, excessive fuel demand, and low efficiency motivated several engineers to apply modifications to improve the steam engine [2]. These machines’ use in human activities was not common because it was considered expensive, with high maintenance costs and low efficiency [3]. The initiative to improve the machine began with searching for new fuels that would offer increased power to the pistons [4]. The use of hydrogen with oxygen in François Isaac de Rivaz’s internal combustion engine was among the first research conducted in this field [5]. This machine’s development led to research in 1851 on pistons fabricated out of cast iron by Barsanti and Felice Matteucci, in an engine where valves were already used to intake and expel gases. This type of engine started combustion using an electric spark. In 1859 and 1860, engineer Jean Joseph Étienne Lenoir built an engine fitted to the Hippomobile. This engine used benzene as fuel [4]. The development of various engines, such as George Brayton’s engine in 1872 [4] or Nikolaus August Otto’s engine in 1877 [6], formed the basis for the implementation of internal combustion engines in cars used as means of transport [7]. However, the engineer Rudolf Diesel, based on Nicolas Léonard Sadi Carnot’s machine, built a combustion engine in 1880 that worked from air and fuel explosion when a certain pressure was applied [8]. In 1897, the engine was fully developed, producing an efficiency of 27%; in comparison, the efficiency of the steam engine was only 10% [9]. Although this engine had high efficiency, it had drawbacks, such as its noise, heavy weight, and problems with the injection system, which presented obstacles to developing the engine in vehicles [10]. The implementation of a rotary pump in 1950 by Vernon Roosa in diesel engines boosted diesel engines’ use in transportation, construction machinery, power plants, and water pumps [11]. The diesel engine development gained in popularity due to its high torque, higher efficiency, resilience to environmental conditions, and reduced pollutant gases [12].

Henry Ford built the first car in 1896, which used ethanol as fuel. Later, it was replaced by gasoline due to its high energy content [13]. However, biofuels were used until the end of the 19th century in Europe. Peanut oil was the first biofuel used in Rudolf Diesel’s engine in 1897. The presence of various processes to transform coal into fuel, the laws to use ethanol as a fuel, and/or the end of World War II in several European countries displaced the use and development of biofuels in internal combustion engines [14]. Regardless, research was conducted by Walton in 1938 on different biofuels fabricated from palm oil, soybean oil, and cottonseed in a diesel engine, showing a fuel economy of 90–91% compared with diesel, although there were problems with the flow of the biofuel and carbon deposits were formed [15]. By 1944, Martinez de Vedia conducted tests using blends of 20% and 40% linseed oil with fossil diesel. The oil’s lubrication properties, ash, and acidity showed increases over pure diesel, and he also found higher amounts of carbon deposits in the combustion chamber when using blends. Therefore, Martinez proposed decanting the fuel before using injectors and filters to reduce plugging [16]. Later, Fort and Blumberg used cottonseed oil as fuel in an experimental test, obtaining performance similar to that of fossil diesel, with no variation in power output in a test lasting 200 h [17]. In 1991, Hemmerlein conducted studies on rapeseed oil and found that the oil’s physical and chemical properties were similar to those of diesel [18]. Finally, European governments in 1997 modified their plans for the introduction of renewable fuels in their energy market [14].

In general, different experimental investigations [19,20] have shown higher values presented by diesel than by biodiesel when comparing the engine’s thermal efficiency, which increase when the load is raised, whereas the specific fuel consumption of the engine is higher when using biodiesel.

Recently, research carried out in 2015 using blends of 20% Adelfa biodiesel and 80% fossil diesel in a diesel engine produced negative results on performance, combustion characteristics, and emissions due to inadequate air/fuel mixing, and the biofuel had a high viscosity. The solution was to create grooves in the piston head, resulting in higher thermal efficiency and lower specific energy consumption due to better air/fuel mixing. Although carbon monoxide (CO) and hydrocarbon emissions decreased, nitrogen oxide (NOx) emissions increased [21]. Some researchers applied variations in piston head geometry. The toroidal-shaped piston stands out from the other designs, showing higher performance, better combustion, and decreased pollutant emissions [22]. This type of piston (toroidal) has been characterized in several experimental tests by increased thermal efficiency, reduction in certain pollutant gases (hydrocarbons (HC) and carbon dioxide (CO_2_), reduction in specific fuel consumption [23], an appropriate air/fuel mixture [22], and reduction in soot [24]. The piston design is intended to properly mix the air with the fuel at the injection time to cause complete combustion inside the combustion chamber [11].

Thermal machines transform thermal energy (heat) into mechanical energy through temperature variation. Heat is obtained from the combustion process (converting fuel into chemical compounds due to an oxidation process) [25]. The energy in transition is called heat [26], and this can be released from a substance called fuel. The main fuels are hydrocarbons, hydrogen, carbon, oxygen, nitrogen, sulfur, ash, and moisture [26,27]. However, biodiesel is also considered fuel and can be obtained from animal fats, vegetable fats, or waste cooking oils. They are produced through a transesterification process, where they are converted into methyl or ethyl esters derived from fatty acids. This fuel is used either blended or in pure form [28]. Currently, the problems of oil extraction and the generation of large amounts of pollutant emissions from this fuel have led to the search for new methods of production [29]. The increase in fuel consumption by the transport sector has led to the use of biofuels from vegetable oils, waste oils, sugars, and animal fats, which are generally mixed with fossil fuels. This type of fuel, called biodiesel, is one option to replace fossil fuels as it has low toxicity and is biodegradable. It is envisaged that by 2050, 27% of energy requirements will be met by biofuels [30].

In Mexico City, there are Official Mexican Environmental Health Standards that regulate air quality to improve the quality of life of its inhabitants. In 2017, there was a reduction in the admissible suspended particulate matter to preserve health [31], so the government has implemented laws to incorporate renewable and clean energy into the energy sector [32]. Means of transport are responsible for 60% of the total consumption of fossil fuels, being large generators of nitrogen oxides (NOx), suspended particles, and carbon dioxide (CO_2_) [33]. The use of biofuels in the energy sector was shown to be an alternative for energy generation and to offer favorable results for the environment [34]. Mexico is considered to be one of the countries with the resources to generate biofuels from animal fat or used oil [35].

In this study, we aim to redesign the geometry of the piston head of a diesel engine to improve the combustion process and reduce the generation of polluting gases using biodiesel mixtures as fuel. The properties of the fuels used in this research are based on the ASTM D 975 and ASTM PS 121 standards. The base piston is a four-cylinder pickup truck engine that generates 131 hp at 3600 rpm. The research is oriented to biodiesel as means of transport in large cities, such as Mexico City.

The remainder of this paper is structured as follows: Section 2 describes the methodology of the analytical calculations of the thermodynamic characteristics of fossil diesel, biofuel, and their mixtures. The new piston design methodology is also provided. Section 2 describes the process of numerical finite element simulation in the reference piston and the new model. Numerical simulations of pressure and temperature on the piston surface are outlined. Section 3 provides the results obtained from the analytical calculations and from the numerical simulations of the pistons that allowed the evaluation and comparison of both models. An analysis of the mechanical and thermal behavior of the two models is provided by numerical simulation when temperature and pressure are applied to the upper surface. The results are compared with other investigations on biofuels and piston models. Section 4 provides our conclusions and future work.

## 2. Methodology

**Figure 1:**
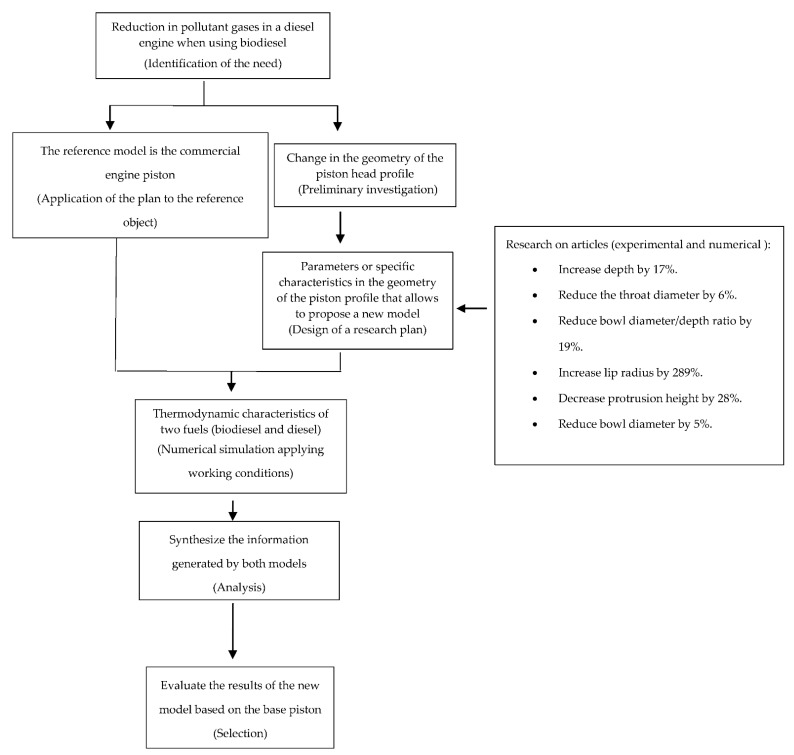
Piston design methodology.

The mechanical engineering design process (Figure 1) was used in this research. This methodology starts from identifying the need; in this case, this was the generation of gases during the combustion process of a diesel engine when using biodiesel as a fuel. Subsequently, a preliminary investigation process was initiated. Some proposals to reduce polluting gases were preheating chambers in the combustion chamber, the use of mixtures of fossil fuel with biodiesel, and the change in geometry of the piston head of the diesel engine.

For this research, the change in the geometry of the piston head was chosen, which has shown positive effects towards reduce polluting gases in the combustion process according to various scientific articles. The thermodynamic characteristics of both fuels were determined to obtain the operating temperature. This parameter served as the basis for the numerical simulation (see Supplementary Material) for the redesign of the piston geometry.

The commercial piston (base piston) of the analyzed diesel engine was taken as a reference. Based on previous studies, specific parameters or characteristics of the geometry of the piston head were obtained that allow the reduction in certain gases. Subsequently, the information obtained from the base piston and from the new design proposal was analyzed. The new design was subjected to mechanical simulations (pressure and temperature) using finite elements to compare the results of both models. The results of the finite element simulations were analyzed and compared with the characteristics of the reference piston.

The results obtained from the volumes, enthalpies, temperatures, and calorific value of the different mixtures are based on the conditions of an adiabatic system, which does not allow the transfer of heat to its surroundings. In addition, it is considered an ideal combustion process, under an ideal diesel cycle, without the intervention of external substances that influence combustion. In the case of fuels (fossil diesel and biodiesel), the calculations are based on properties established by the ASTM D 975 and ASTM PS 121 standards [28] (See Supplementary Material: Table S1. Properties of biodiesel and diesel and Table S2: Chemical composition of fuels). Therefore, for this research, the main objective was to redesign the head geometry of the piston because the design process begins with the modification of the piston geometry and later with the design of other components such as rings, ceramic coatings, heat treatments, and cooling channels.

### 2.1. Numerical Simulation

#### 2.1.1. Numerical Simulation of the Base Piston

**Figure 2:**
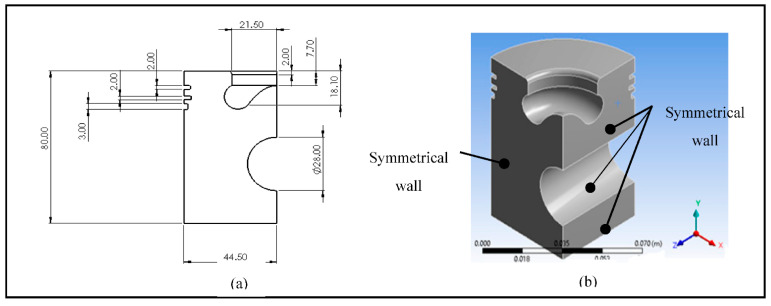
(**a**) Piston dimensions in (mm) and (**b**) piston model.

To conduct this simulation, the dimensions of a pickup truck piston were used (Figure 2a) [36,37]. This model served as the basis for comparison and evaluation with the proposed piston head redesign. The finite element method was used because it offers high-quality results and a wide range of results. Symmetry was applied to the model (on the side walls) because only one-quarter of the piston was developed (Figure 2b). The simulation was performed in Transient Thermal of ANSYS^®^ 19.2 (Ansys Drive, Canonsburg, PA, USA). In this analysis, an isotropic, linear-elastic, homogeneous, and continuous material was considered. The material used was aluminum 2024. Table 1 shows the material’s properties.

**Figure 3:**
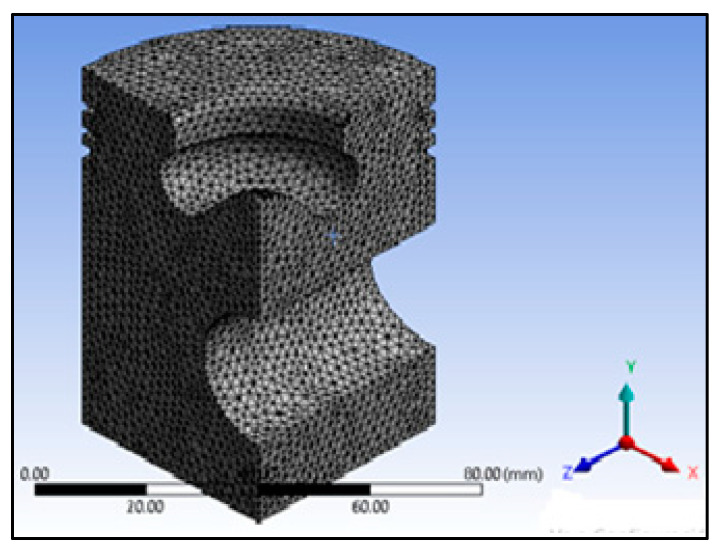
Meshed model of the base piston.

Figure 3 shows the meshed model after applying high-order elements (intermediate nodes). We considered forced convection to transfer heat between the combustion gases and the valves, cylinder walls, and the piston during the diesel cycle [40]. The heat transfer was used on the top surface of the piston model with a coefficient of $h=450\;\frac{W}{m^{2}\;{}^{\circ}C}$ [41] for 1 s, assuming an initial temperature of 22 °C.

A compressive pressure of 3.1 MPa [42] on the piston’s top surface was used to determine the stresses inside the piston. In this simulation, the pin’s area was restricted to 6 degrees of freedom (displacement in x, y, and z directions; rotation in xy, xz, and yz planes). Figure 3 shows the piston’s meshed model, and the simulation was performed in Static Structural of ANSYS 19.2.

#### 2.1.2. Proposed Redesign for the Piston Head

**Figure 4:**
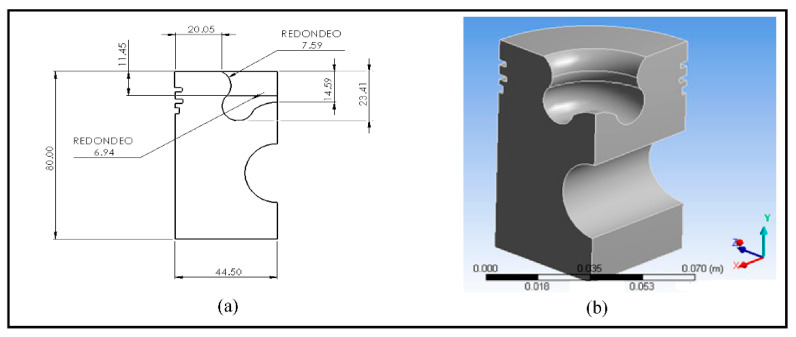
(**a**) Dimensions of the redesign of the piston head (mm); (**b**) model of the redesign in Transient Thermal.

For the piston redesign process, the information in Table 2 was considered. In addition, the cone angle must range from 150° to 160° [43]. In this proposed design, the dimensions of the diameter, the outside diameter of the piston, and the piston rings’ positions were unchanged. Changes were applied to the profile of the piston head to keep the compression ratio constant and confirm that the possible variations that occurred were due to changes in the piston geometry [19]. Figure 4 shows the dimensions of the modifications to the piston profile geometry and the model used to perform the numerical simulations. Aluminum 2024 was used for this model.

In this simulation, the proper diesel temperature $h=450\;\frac{W}{m^{2}\;{}^{\circ}C}$ [41] with an initial temperature of 22 °C. The simulation was carried out for 1 s.

**Figure 5:**
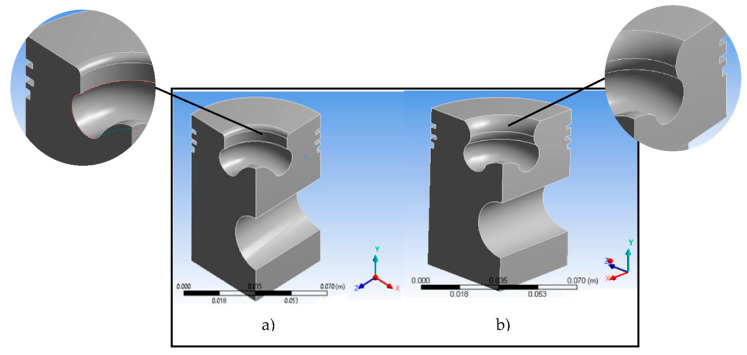
Model comparison: (**a**) base model; (**b**) redesigned piston geometry.

To determine stresses within the piston head redesign, a compressive pressure of 3.1 MPa [42] was used on the piston’s top surface. Similarly, the pin’s area was constrained to 6 degrees of freedom (displacement in x, y, and z directions; rotation in xy, xz, and yz planes). The simulation was performed in Static Structural of ANSYS 19.2. Figure 5 shows a comparison of the base model and the proposed piston geometry.

## 3. Results and Discussion

### 3.1. The Thermodynamic Characteristics of Diesel and Biodiesel

Table 2 and Table 3 show the results of the combustion volumes of various biodiesel blends. The results showed a decrease in gases with increasing biodiesel content in the mixtures, demonstrating the advantages of using this biofuel in diesel engines.

The results of other studies on combustion simulations using diesel and biofuels showed a reduction in pollutant gases, with a 23% reduction in H_2_O, 13% in CO_2_, 21% in O_2_, and 21% in N_2_ compared with commercial diesel [48]. This demonstrates the reduction in gases when using biodiesel in combustion [49]. The results of some experimental tests with biodiesel and diesel showed a decrease of 43% in CO_2_, 55% in suspended particles, and 54% in hydrocarbons compared with diesel, but there was a 6% increase in nitrogen oxides (NOx) [28]. Table 3 and Table 4 show the decrease in combustion products with increasing biodiesel content in the blend, consistent with previously mentioned research, and demonstrates the advantage of using biodiesel as a fuel.

**Figure 6:**
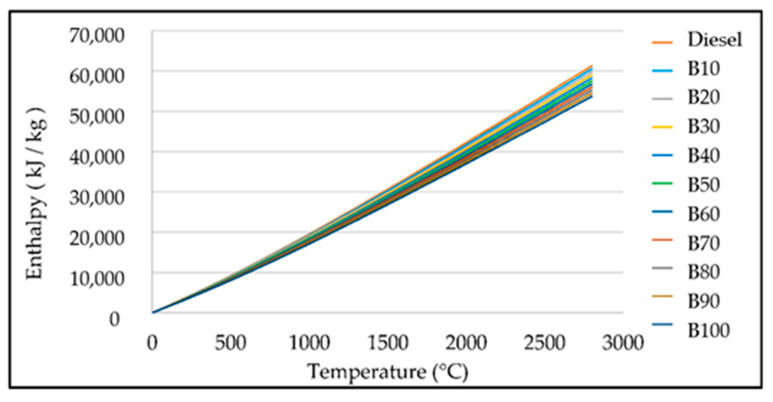
Enthalpy of combustion products for diesel and biodiesel.

Figure 6 shows the enthalpy generated by each proposed fuel mixture. The decrease in enthalpy is observed when using a higher biodiesel content in the mixture, responsible for each mixture’s chemical composition and factors such as temperature and pressure.

The amounts of hydrogen and carbon are important since these elements are considered a significant source of energy [50]. Oxygen in fuels has an antiknock capacity that reduces the heat that can be generated during the combustion process [51]. Although the sulfur content for this research was considered null, it is an undesirable element in fuels because it damages the atmosphere by the emission of sulfur dioxide (SO_2_). In some cases, sulfur can improve the lubricity of engines’ fuels [52]. Biodiesel contains less carbon and hydrogen, which reduces the power generation of this fuel. Figure 6 shows the tendency of the combustion products’ enthalpy to decrease when the biodiesel in the mixtures increases.

Table 5 shows the calorific value obtained from each mixture. The calorific value decreased when the content of biodiesel in the mixture increased. The calorific value of biodiesel was reduced by approximately 13% compared with fossil diesel, representing greater fuel consumption in the engine by offering less power.

In B100, a calorific value of 37,150 kJ/kg [53] was obtained. In other research, biodiesel (oleander oil) and fossil diesel were found to have calorific values of 42,650 and 44,120 kJ/kg, respectively [19]. Based on the ASTM D240 standard, diesel and biodiesel have values of 44,800 and 40,500 kJ/kg, respectively [20]. The use of biofuel (Higuerilla oil) in biodiesel, compared with Mexican diesel with a value of 44,040 kJ/kg, had a lower calorific value due to the higher oxygen content in the fuel, which represents lower engine power [49] and an increase in fuel consumption [54]. Table 5 shows the decreasing behavior in the upper and lower calorific values with increasing biodiesel content in the blends; the behaviors and values obtained from Table 5 are similar to those acquired in previous research.

Table 6 shows the adiabatic flame temperatures of each mixture; the decrease in temperature of less than 1% of biodiesel compared with fossil diesel can be observed. These results do not represent large changes, so the variation between them is considered insignificant.

Table 7 shows the combustion temperatures of both fuels, of which only the applicable temperature represents the condition in which the piston head is exposed. This temperature coincides with those reported in various books and manuals [55]. In addition, the temperature does not reach melting temperature, which would cause damage to the combustion chamber [56].

In some engines, the combustion chamber’s gas temperature is 2200 °C [57], although it can also vary from 700 to 900 °C inside the chamber [58]. More detailed investigations showed that the combustion chamber can reach 250 °C, the chamber walls 150 to 200 °C, and the piston head 250 to 300 °C [55]. Factors such as more excess air within the combustion [59] and the fuels’ chemical composition influence the temperature generated in the combustion process. The various conditions to which the engine may be subjected result in various operating temperatures, although it is important to consider the limits of conditions that can be withstood by an engine to function correctly. Table 7 shows temperatures that can be generated in the combustion chamber, which coincide with those previously reported—even the calculated useful temperature (diesel and biodiesel) is less than 66% of the melting temperature of the material, a parameter that ensures its correct operation in the engine [56].

### 3.2. Numerical Results for Diesel and Biodiesel Temperature Application on the Base Piston

**Figure 7:**
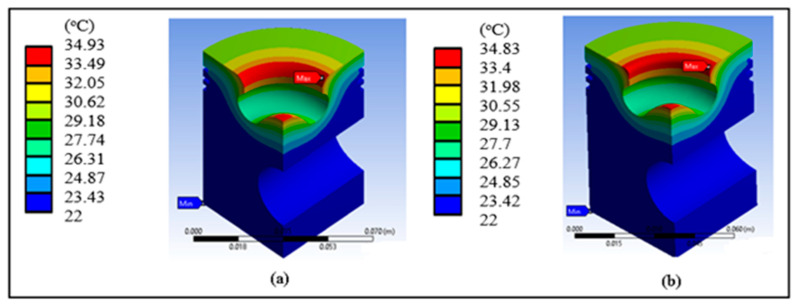
Surface temperature of the base piston with (**a**) diesel and (**b**) biodiesel.

**Figure 8:**
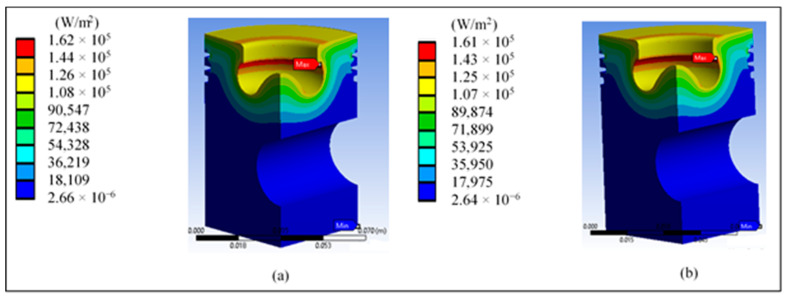
Total heat flow in the base piston with (**a**) diesel and (**b**) biodiesel.

Figure 7 shows the temperature inside the piston, which is only concentrated in the radius of the lip and at the tip; the values do not reach the melting temperature. Figure 8 shows the total heat flow in the base piston. The highest values are concentrated only in the lower part of the lip radius, an area where the heat of combustion is expected to be expelled.

### 3.3. Numerical Results of Determining Stresses in the Base Piston

**Figure 9:**
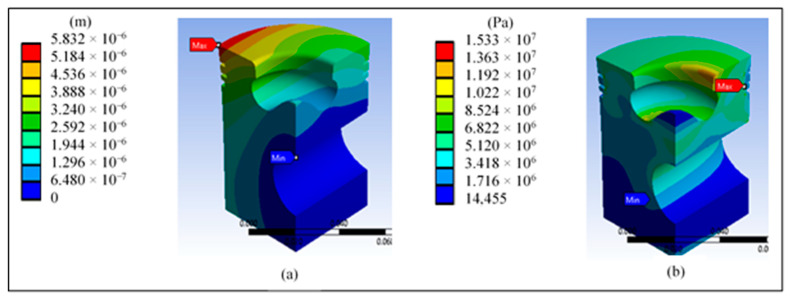
Results at the base piston’s head: (**a**) total displacement and (**b**) von Mises stress.

Figure 9a shows the total displacement of the piston when applying pressure. The outer edges show the highest values, which coincides with various consulted sources and justifies the conical shape in the upper part of the piston. Figure 9b shows that the highest von Mises stress is concentrated in the third ring area. However, the maximum value of 15.33 MPa does not represent a risk compared with the yield stress of 325 MPa of the proposed material.

**Figure 10:**
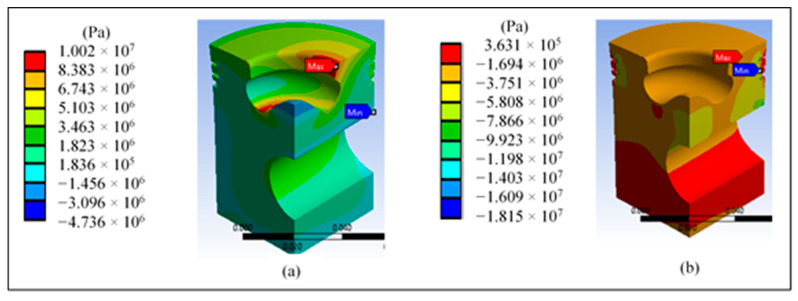
Results at the base piston head: (**a**) maximum and (**b**) minimum principal stress.

Figure 10a shows a higher maximum tensile stress in the area of the lip radius, reaching a value of 10 MPa. Figure 10b shows the principal stress of 18.15 MPa in the area near the piston surface and the area of the pin.

**Figure 11:**
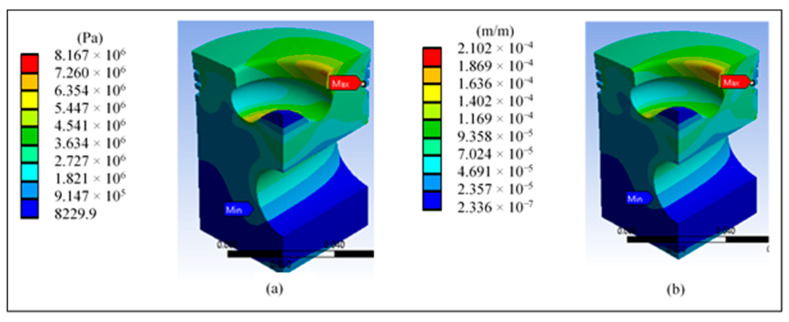
Results on the head of the base piston: (**a**) maximum shear stress and (**b**) total unit strain.

Figure 11a shows a maximum shear stress of 8.16 MPa in the third ring area, which does not represent a risk against the material’s yield stress. Figure 11b shows the maximum strain in the third ring area, which is cause for concern in this area due to the simulation’s high values.

Table 8 shows the maximum and minimum results from the simulations performed on the base piston.

### 3.4. Numerical Results of Diesel Temperature on the Piston Redesign

**Figure 12:**
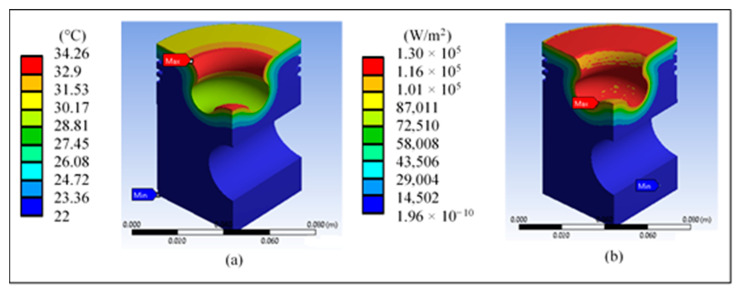
Results of the piston head redesign: (**a**) piston temperature and (**b**) total heat flow.

Figure 12a shows the temperature inside the modified piston, concentrated in the radius of the lip and the tip, and the higher values do not reach the melting temperature of the material. Figure 12b shows the total heat flow in the modified piston. The highest values are concentrated in almost the entire upper area of the piston, except for the lip rake. This effect may cause problems in the expulsion of the heat flow.

### 3.5. Numerical Results for Determining Stresses in the Piston Crown Redesign

**Figure 13:**
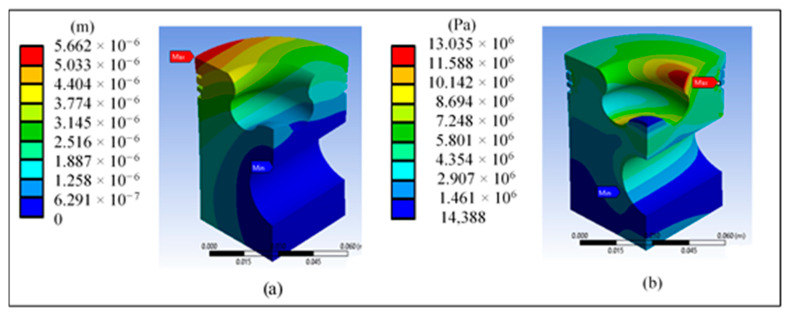
Piston head redesign results: (**a**) total displacement and (**b**) von Mises stress.

Figure 13a shows the total displacement of the piston when applying pressure; the outer edges show the highest values and the maximum value obtained does not represent problems. Figure 13b shows that the highest von Mises stress is concentrated in the third ring area and the lip radius. The maximum value of 13.035 MPa does not represent a risk compared with the yield stress of 325 MPa of the proposed material.

**Figure 14:**
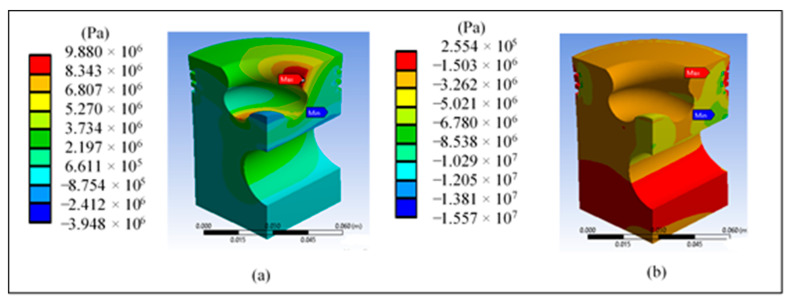
Results of the piston head redesign: (**a**) maximum and (**b**) minimum principal stress.

Figure 14a shows a higher maximum tensile stress in the area of the lip radius, reaching a value of 9.88 MPa. However, Figure 14b shows a minimum principal stress of −15.57 MPa in the area near the piston surface and the area of the pin.

**Figure 15:**
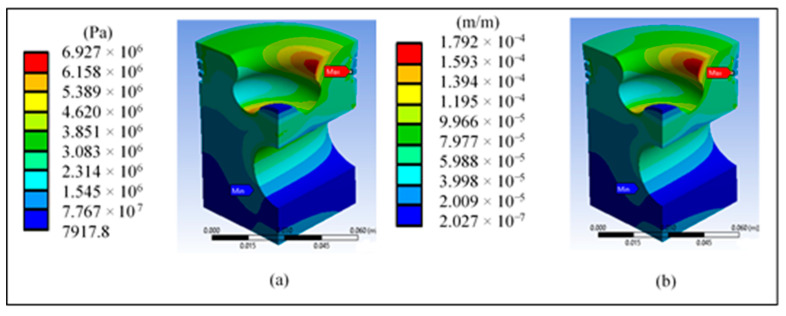
Piston crown redesign results: (**a**) maximum shear stress and (**b**) total unit strain.

Figure 15a shows a maximum shear stress of 6.92 MPa in the third ring area and in the radius of the lip, which does not represent a risk against the yield stress of the material. Figure 15b shows a maximum strain in the third ring area and in the area of the lip radius, which represents a greater risk in this area due to the high values generated in the simulations.

In similar simulations performed on an aluminum alloy piston (aluminum–silicon), applying a temperature of 300 °C on the surface with a coefficient of 230 W/m^2^·K with various temperatures and coefficients in different areas of the piston showed similar behavior to that obtained in the simulations, i.e., the maximum points were centered on the piston tip and the lip radius, reaching a value of 285 °C [56].

In simulations to obtain stresses and deformations, a pressure of 1.16 MPa was applied to the piston surface using aluminum G4658, resulting in a von Mises stress of 80.64 MPa in the pin area, with a maximum total deformation of 9.06 × 10^−6^ m located at the top center of the piston [60]. In a similar simulation, a pressure of 3 MPa was applied to the piston surface at a temperature of 300 °C with a coefficient of 5 × 10^−6^ W/mm^2^ °C with eutectic material (AlSi12CuMgNi); the results showed a von Mises stress of 97.9 MPa in the area near the pin, with a maximum unit deformation of 1.3396 mm/mm and a maximum heat flux of 53.02 × 10^5^ W/m^2^ in the upper central area of the piston head [61]. There is usually a clearance between the combustion chamber and the piston that allows for proper lubrication of the chamber’s mechanism. In the piston skirt area, it can be 0.15 to 0.35 × 10^−4^ m [62] or up to 0.5 × 10^−4^ m [63]. 

The results obtained from Table 8 and Table 9 show 10% of the melting temperature. The heat flow behavior presented by the piston redesign showed drawbacks, as the heat is expected to be directed through the bowl radius through the chamber walls. The expulsion of heat through the top of the piston represents the admission and expulsion of heat in each cycle, an effect that can cause damage to the piston. 

The displacements obtained from the simulations are smaller than the clearances that can exist between the combustion chamber and the piston. The von Mises stress only represents 4% of the yield stress of the material. The principal stresses present higher values in compression than in tension, whereas the maximum shear stress only represents 4% of the yield shear stress of the material.

The redesign of a diesel engine piston involves changing several operating conditions and factors that can affect the performance of the engine. The design of various diesel engines depends on the purpose of the application, so this purpose must be considered. The biofuel supplied for its operation is another factor of importance because its properties and behavior depend on the geographical area of extraction and the internal and external conditions of the engine. This research was limited to the analysis of a pickup truck. However, the results and analysis obtained will be applied later to various diesel engines to replace the use of fossil fuels.

This paper presented numerical simulation tests to characterize the stress and strain in piston engines and their feasibility, mainly from the design viewpoint. We established a material selection that focuses on the data modeling aspects of the problem, where data are presented in charts. In the literature, a sensitive mesh analysis is not always necessary when anisotropic and nonlinear performance are not considered. In this study, we use isotropic, continuous, and homogeneous data in the numerical analysis [64].

## 4. Conclusions

The thermodynamic characteristics of the fuels produced reductions in combustion products when using biodiesel as a fuel. Fossil diesel showed higher total enthalpy generation than biodiesel, including a higher calorific value; the highest adiabatic flame temperature was found for fossil diesel. However, the difference in the results of the two fuels is less than 13%. The use of biodiesel as a fuel produced results similar to those of diesel, offering a reduction in pollutant gases and a new alternative to generate energy, as biodiesels are fabricated from vegetable or waste oils, sugars, or animal fats.

The behaviors and values obtained from the numerical simulations of both models were similar, including the redesign of the piston head. However, the redesigned piston showed a higher heat flow in the piston’s upper area, which affects the piston because the heat flow is expected to be expelled by the bowl radius toward the walls of the combustion chamber. Notably, the application of piston rings, ceramic coatings, heat treatments, and cooling channels that affect piston behavior were not considered in this research. The stresses, deformations, and displacements resulting from the simulation of both pistons were similar. The values are considered acceptable, although the maximum values tend to occur in the areas close to the piston rings and the bowl lip, which is why greater attention should be paid to this area as the rings play an important function inside the combustion chamber.

## Figures and Tables

**Table 1:** Material’s properties [38,39].

Parameter	Aluminum Alloy 2024
Young’s modulus (MPa)	73,000
Poisson’s ratio	0.33
Density (kg/m^3^)	2770
Tensile yield strength (MPa)	325
Ultimate tensile strength (MPa)	470
Thermal conductivity (W/m °C)	120
Melting point (°C)	500–638

**Table 2:** Technical simulation considerations.

Parameter	Increase	Decrease	Effect
Bowl depth	X		Reduce emissions of soot, hydrocarbons (HC), carbon monoxide (CO), nitrogen oxides (NOx), and specific fuel consumption [24]
Throat diameter		X	Reduce specific fuel consumption and increase crushing [23]
Bowl diameter/depth ratio		X	Increase crushing, improve air/fuel mixture, and generate higher efficiency [44]
Lip radius	X		Increase performance and reduce the generation of polluting emissions [22]
Outside diameter		X	Decrease throat diameter ratio to increase swirl and increase turbulence [45]
Protrusion height		X	Reduce emissions of nitrogen oxides (NOx) [46]
Bowl diameter		X	Reduce the generation of smoke and generate greater crushing [47]

**Table 3:** Combustion gases [48].

Content (m^3^/kg)	B0	B10	B20	B30	B40	B50	B60	B70	B80	B90	B100
VO	11.18	11.03	10.88	10.72	10.57	10.42	10.27	10.12	9.96	9.81	9.66
VO_2_	2.34	2.31	2.28	2.25	2.22	2.19	2.15	2.12	2.09	2.06	2.03
VCO_2_	1.62	1.60	1.58	1.56	1.54	1.53	1.51	1.49	1.47	1.45	1.43
VSO_2_	0.00	0.00	0.00	0.00	0.00	0.00	0.00	0.00	0.00	0.00	0.00
VN_2_O	8.83	8.71	8.59	8.47	8.35	8.23	8.11	7.99	7.87	7.75	7.63
VH_2_O	1.62	1.61	1.60	1.58	1.57	1.56	1.54	1.53	1.51	1.50	1.49
VRO_2_	1.62	1.60	1.58	1.56	1.54	1.53	1.51	1.49	1.47	1.45	1.43

**Table 4:** Gases in the stack.

Content (m^3^/kg)	B0	B10	B20	B30	B40	B50	B60	B70	B80	B90	B100
V_R2_^O^	9.39	9.26	9.14	9.01	8.88	8.75	8.62	8.5	8.37	8.24	8.11
V_H2O_^O^	1.63	1.62	1.6	1.59	1.58	1.56	1.55	1.54	1.52	1.51	1.50
VGas	12.64	12.48	12.32	12.16	12.00	11.84	11.68	11.52	11.36	11.20	11.04

**Table 5:** Higher heating values (HHV) and lower heating values (LHV) of fuels.

Content (kJ/kg)	B0	B10	B20	B30	B40	B50	B60	B70	B80	B90	B100
HHV	45,734	45,150	44,567	43,983	43,400	42,816	42,233	41,649	41,006	40,482	39,899
LHV	42,809	42,248	41,687	41,126	40,565	40,004	39,443	38,882	38,321	37,760	37,199

**Table 6:** Adiabatic flame temperature of fuels.

Content	B0	B10	B20	B30	B40	B50	B60	B70	B80	B90	B100
Temp. (°C)	2024	2023	2021	2020	2019	2017	2016	2014	2013	2012	2010

**Table 7:** Combustion temperatures.

Temperature	Diesel	Biodiesel
Adiabatic flame temperature (°C)	2024	2010
Actual flame temperature (°C)	971	965
Useful temperature (°C)	291	289

**Table 8:** Results of the numerical simulations on the base piston.

Simulation	Diesel		Biodiesel	
	Max.	Min.	Max.	Min.
Piston temperature (°C)	34.931	22	34.835	22
Total heat flux (W/m^2^)	1.629 × 10^5^	2.667 × 10^−6^	1.617 × 10^5^	2.649 × 10^−6^
**Stress**	**Max.**		**Min**	
Total displacement (m)	5.832 × 10^−6^		0	
von Mises stress (MPa)	15.332		14.455 × 10^−3^	
Maximum principal stress (MPa)	10.023		−4.736	
Minimum principal stress (MPa)	0.363		−18.153	
Maximum shear stress (MPa)	8.167		8.229 × 10^−3^	
Total unit strain (m/m)	2.102 × 10^−4^		2.336 × 10^−7^	

**Table 9:** Simulation results on the piston head redesign.

Simulation	Redesigned Piston	
	Max.	Min.
Piston temperature (°C)	34.26	22
Total heat flux (W/m^2^)	1.305 × 10^5^	1.962 × 10^−^^10^
Total displacement (m)	5.662 × 10^−^^6^	0
von Mises Stress (MPa)	13.035	14.388 × 10^−^^3^
Maximum principal stress (MPa)	9.880	−3.948
Minimum principal stress (MPa)	0.255	−15.574
Maximum shear stress (MPa)	6.927	7.917 × 10^−^^3^
Total unit strain (m/m)	1.792 × 10^−^^4^	2.027 × 10^−^^7^

## Data Availability

Not applicable.

## Supplementary Materials

The following are available online at https://www.mdpi.com/article/10.3390/ma14112812/s1, Suptopic: 2.1. Thermodynamic characteristics, Table S1: Properties of biodiesel and diesel, Table S2: Chemical composition of fuels.
