# Supplementary material for: Redesign of a Piston for a Diesel Combustion Engine to Use Biodiesel Blends
Source: Materials (Basel). 2021 May 25;14(11):2812. doi: 10.3390/ma14112812 (PMC8197454; doi:10.3390/ma14112812)
Supplement: Supplementary file 1 [file materials-14-02812-s001.zip › materials-1187656-supplementary.pdf]

materials

MDPI

Article

Redesign of a Piston for a Diesel Combustion Engine to Use Biodiesel Blends

Jorge Israel Noriega Lozano [1], Juan Carlos Paredes Rojas [2,*], Beatriz Romero Ángeles [1], Guillermo Urriolagoitia Sosa [1], Belén Alejandra Contreras Mendoza [1], Christopher Rene Torres San Miguel [1], Georgiy Polupan [1] and Guillermo Manuel Urriolagoitia Calderón [1]

1 Instituto Politécnico Nacional, Escuela Superior de Ingeniería Mecánica y Eléctrica, Unidad Profesional Adolfo López Mateos, Gustavo A. Madero, Col. Lindavista, C.P. 07738 Ciudad de México, Mexico; jnoriegalozano@gmail.com (J.I.N.L.); BROMEROA@ipn.mx (B.R.Á); guiurri@hotmail.com (G.U.S.); bele02mendoza@gmail.com (B.A.C.M.); ctorress@ipn.mx (C.R.T.S.M.); gpolupan@ipn.mx (G.P.); gurriolagoitiac@ipn.mx (G.M.U.C.)

2 Instituto Politécnico Nacional, Centro Mexicano para la Producción más Limpia, Acueducto de Guadalupe S/N, La laguna Ticomán, C.P. 07340 Ciudad de México, Mexico

* Correspondence: jparedes@ipn.mx

Abstract: Biofuels represent an energy option to mitigate polluting gases. However, technical problems must be solved, one of them is to improve the combustion process. In this study, the geometry of a piston head for a diesel engine was redesigned. The objective was to improve the combustion process and reduce polluting emissions using biodiesel blends as the fuel. The methodology used was the mechanical engineering design process. A commercial piston (base piston) was selected as a reference model to assess the piston head's redesign. Changes were applied to the profile of the piston head based on previous research and a new model was obtained. Both models were evaluated and analyzed using the finite element method, where the most relevant physical conditions were temperature and pressure. Numerical simulations in the base piston and the new piston redesign proposal presented similar behaviors and results. However, with the proposed piston, it was possible to reduce the effort and the material. The proposed piston profile presents adequate results and behaviors. In future, we suggest continuing conducting simulations and experimental tests to assess its performance.

Keywords: biodiesel; piston; diesel engine; numerical simulation; finite element method

Citation: Lozano, J.I.N.; Rojas, J.C.P.; Ángeles, B.R.; Sosa, G.U.; Mendoza, B.A.C.; Miguel, C.R.T.S.; Polupan, G.; Calderón, G.M.U. Redesign of a Piston for a Diesel Combustion Engine to Use Biodiesel Blends. *Materials* **2021**, *14*, 2812. https://doi.org/10.3390/ma14112812

Academic Editor: Claudio Giardini

Received: 31 March 2021
Accepted: 18 May 2021
Published: 25 May 2021

Publisher's Note: MDPI stays neutral with regard to jurisdictional claims in published maps and institutional affiliations.

Copyright: © 2021 by the authors. Licensee MDPI, Basel, Switzerland. This article is an open access article distributed under the terms and conditions of the Creative Commons Attribution (CC BY) license (http://creativecommons.org/licenses/by/4.0/).

Thermodynamic characteristics

In order to calculate the thermodynamic characteristics of fossil diesel and biodiesel, was necessary to determine the chemical composition of the fuels. A 0% sulphur content was considered due to the low sulphur content. The physical and chemical properties of both fuels are presented in Table S1 and their chemical composition is shown in Table S2.

Subsequently, the combustion products resulting from both fuels were calculated. For these calculations, incomplete combustion, the addition of lubricating oil, additives or sulphur in the combustion process were neglected [34]. The following calculations were valid for 1 kg of fuel, under standard pressure conditions of 760 mm Hg at a temperature of 0°C. To calculate the theoretical amount of air required for 1 kg of fuel, the following formula shall was used [35]:

$$V^{o} = 0.0889(C + 0.375\ S) + 0.265\ H - 0.0333\ O \tag{1}$$

The letters C, S, H and O refer to the content (%) of Carbon, Sulphur, Hydrogen and Oxygen in the fuel. To determine the theoretical amount of Oxygen, where r_{O_2}=1 .42$\frac{kg}{m^3}$ is the density of Oxygen [36], it is calculated by:

$$V^{0}_{O_2} = 1.866\ \frac{C}{100} + 0.7\frac{S}{100} + 5.55\frac{H}{100} - \frac{O}{100^{*}(r_{O_2})} \tag{2}$$

The formula for determining the volume of the products of combustion of carbon dioxide is:

$$V_{CO_2} = 1.86\frac{C}{100} \tag{3}$$

To calculate the theoretical volume of Nitrogen is:

$$V^{0}_{N_2} = 0.79\ (V^{o}) \tag{4}$$

To determine the theoretical volume of the vapours is:

$$V^{0}_{H_2O} = 0.111\ H + 0.0161\ (V^{o}) \tag{5}$$

The volume of tri-atomic gases is calculated by:

$$V_{RO_2} = 1.86\ \frac{C + 0.375\ S}{100} \tag{6}$$

In order to develop complete combustion inside the combustion chamber, an extra amount of air is added to the process, called excess air [23]. For this calculation, an excess air coefficient (α) of 1.05 was used, because a higher air excess increase the generation of gases such as oxygen (O_2), nitrogen (N_2) [37], carbon dioxide (CO_2) [38], nitrogen oxides [39] and soot [40]. The actual volume of diatomic gases depending on the excess air coefficient is:

$$V_{R_2} = V^{0}_{N_2} + (\alpha - 1)\ V^{o} \tag{7}$$

To calculate the real vapor volumes considering α >1 is:

$$V_{H_2O} = V^{0}_{H_2O} + 0.0161\ (\alpha - 1)V^{o} \tag{8}$$

The volume of gases to the stack is calculated:

$$V_{gas} = V_{RO_2} + V_{R_2} + V_{H_2O} \tag{9}$$

To calculate the enthalpy (H) of each gas as a function of its volume, considering combustion at constant pressure, Cp (Appendix 1) and varying the temperature (°C):

$$H_{CO_2} = V_{CO_2}{}^{*}\ Cp_{CO_2}{}^{*}\ T \tag{10}$$

$$H_{H_2O} = V_{H_2O}{}^{*}\ Cp_{H_2O}{}^{*}\ T \tag{11}$$

$$H_{N_2} = V_{N_2}{}^{*}\ Cp_{N_2}{}^{*} \tag{12}$$

$$H_{gas} = H_{CO_2} + H_{H_2O} + H_{N_2} \tag{13}$$

$$H_{air} = V^{o}\ {}^{*}\ Cp_{air}{}^{*}\ T \tag{14}$$

$$H_{excess\ air} = H_{air}{}^{*}\ (\alpha\ -1) \tag{15}$$

$$H_{TOTAL} = H_{gas} + H_{excess\ air} \tag{16}$$

To determine the higher heating value (HHV) and the lower heating value (LHV) of fuels the following formulas were used [41]:

$$HHV = 338(C) + 1256(H) - 109\ (O\text{-}S) \tag{17}$$

$$LHV = PCS - 25\ [9(H)] \tag{18}$$

The maximum temperature obtained within the combustion process is called adiabatic flame temperature. This temperature is essential in the design of combustion chambers [24] (material selection and stress calculation [23]). Based on the calculations of the fuels' PCI, these values were then looked up in the previously calculated total enthalpy results, and through interpolation, the temperature at which they are found was determined.

Table S1. Properties of biodiesel and diesel [26].

Properties	DIESEL	BIODIESEL
Norm	ASTM D975	ASTM PS 121
Kinetic viscosity at 40°C	1.3–4.1	1.9–6
Specific gravity at 15.55 ° C	0.85	0.88
Density (lb/gal)	7.079	7.328
Carbon %	87	77
Hydrógen %	13	12
Oxygen %	0	11
Sulfur %	0.05	0.0024
Flash point (° C)	60–80	100-–70
Cetane number	-35–15	-15a–10
Stoichiometric ratio (air / fuel)	40–55	48–65

Table S2. Chemical composition of fuels [26].

Composition	Diesel	Biodiesel
Carbon %	87	77
Hydrogen %	13	12
Oxygen %	0	11
Sulfur %	0	0
Total %	100	100

The adiabatic flame temperature was obtained under ideal combustion conditions [38], although the actual flame temperature is regularly 48 to 75% of the adiabatic flame temperature [42]. For this analysis, 48% of the calculated temperature was used. In diesel engines, of the total energy produced in the combustion process, only about 30% is directed to the flue gas [43], and this is the helpful temperature considered for application in the numerical simulation.
